# RelAgent: a multi-agent solution for molecular relationship grounding

**DOI:** 10.1093/bioinformatics/btag541

**Published:** 2026-07-23

**Authors:** Rubing Chen, Jiaxin Wu, Chen Jason Zhang, Xiao-Yong Wei, Qing Li

**Affiliations:** Department of Computing, The Hong Kong Polytechnic University, Hung Hom, Hong Kong SAR; The Hong Kong Polytechnic University Shenzhen Research Institute, Shen Zhen, Guangdong Province, China; Department of Computing, The Hong Kong Polytechnic University, Hung Hom, Hong Kong SAR; The Hong Kong Polytechnic University Shenzhen Research Institute, Shen Zhen, Guangdong Province, China; Department of Computing, The Hong Kong Polytechnic University, Hung Hom, Hong Kong SAR; The Hong Kong Polytechnic University Shenzhen Research Institute, Shen Zhen, Guangdong Province, China; Department of Computing, The Hong Kong Polytechnic University, Hung Hom, Hong Kong SAR; The Hong Kong Polytechnic University Shenzhen Research Institute, Shen Zhen, Guangdong Province, China; Sichuan University, Chengdu, Sichuan Province, China; Department of Computing, The Hong Kong Polytechnic University, Hung Hom, Hong Kong SAR; The Hong Kong Polytechnic University Shenzhen Research Institute, Shen Zhen, Guangdong Province, China

## Abstract

**Motivation:**

Molecular captions, patents, and medicinal-chemistry notes describe substructures and their relations in natural language, whereas computational models operate on formal representations such as SMILES. Bridging this semantic–structural gap is important for patent interpretation, structural relationship analysis, and controllable molecular editing, yet current large language models struggle to ground textual references to precise molecular components.

**Results:**

We propose RelAgent, a cooperative multi-agent framework for molecular relationship grounding. RelAgent decomposes the task into three interpretable stages: entity extraction, substructure localization, and ontology-guided relationship reasoning, and then uses verifier agents to rank structurally plausible candidates. This design supports fine-grained reasoning over molecular substructure and substantially improves performance on the MolGround benchmark. RelAgent achieves 81.4% entity-extraction F1, 56.0% exact-match localization F1, and 54.6% relationship F1 on an open-source LLaMA3.1-8B model, improving the REL F1 from 0.1% to 54.6% and exceeding the vanilla Gemini-3.1-Pro baseline in our experiments. These results indicate that agentic, structure-aware reasoning is a practical direction for interpretable molecular understanding in bioinformatics.

**Availability and implementation:**

The source code for RelAgent is available at https://github.com/Anya-RB-Chen/RelAgent

## 1 Introduction

**Figure 1:**
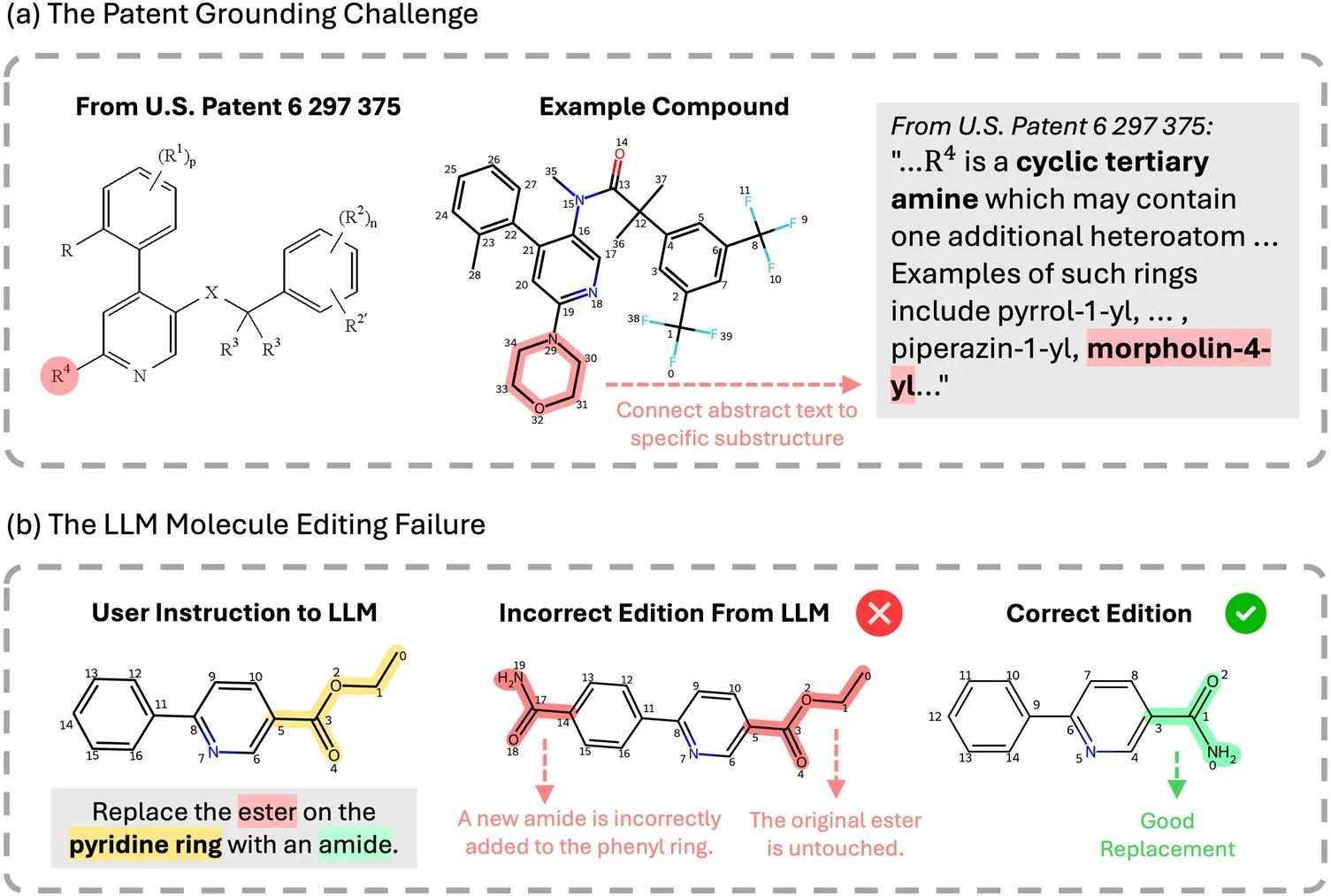
Two core challenges in molecular grounding: (a) Mapping abstract descriptions from a chemical patent to a concrete substructure. (b) The failure of a standard LLM to execute a precise editing command due to its inability to correctly localize the target substructure.

Understanding the intricate relationships between a molecule’s constituent substructures is critical for scientific progress in fields like drug discovery and materials science (Liu *et al*. 2021, Chakraborty *et al*. 2023, Bran and Schwaller 2024). The function of a molecule is not merely a sum of its parts, but emerges from the precise way these components are arranged and connected (Kuhn 1972, Lehn 2013). The necessity for an intelligent model that can master this understanding is critical from both human and agent perspectives. From the human perspective, this challenge is starkly evident when trying to interpret the complex and legally dense language of chemical patents. As illustrated in Fig. 1a, a patent may define a vast chemical space using Markush structures, where a single placeholder like R^4^ can represent dozens of possible fragments. For example, in a patent for neurokinin-1 receptor antagonists, R^4^ is abstractly defined as a “cyclic tertiary amine,” with examples including “morpholin-4-yl” and “piperazin-1-yl” among others (U.S. Patent 62,97,375) (Bös *et al.* 2001). An intelligent assistant must be able to parse this abstract legal language and accurately ground it to the specific morpholino substructure in a concrete molecule, a task that is a critical bottleneck for novelty and freedom-to-operate analysis (Kosonocky *et al*. 2024, Vangala *et al*. 2024). From an agent’s perspective, this grounding failure is even more pronounced (Fig. 1b). While Large Language Models (LLMs) can process chemical text, they fail at performing the structured edits essential for tasks like lead optimization (Udegbe *et al*. 2024). An LLM cannot reliably execute a command such as “*replace the ester on the pyridine ring with an amide*” (Ye 2024, Averly *et al*. 2025). Because the model is unable to ground the textual phrase “the ester on the pyridine ring” to the specific atoms and bonds in the molecular graph, it may incorrectly modify a different functional group or even the wrong aromatic ring entirely. This failure to localize textual references prevents LLMs from enabling truly interactive and controllable molecular design (Huang *et al*. 2024, Liu *et al*. 2024).

**Figure 2:**
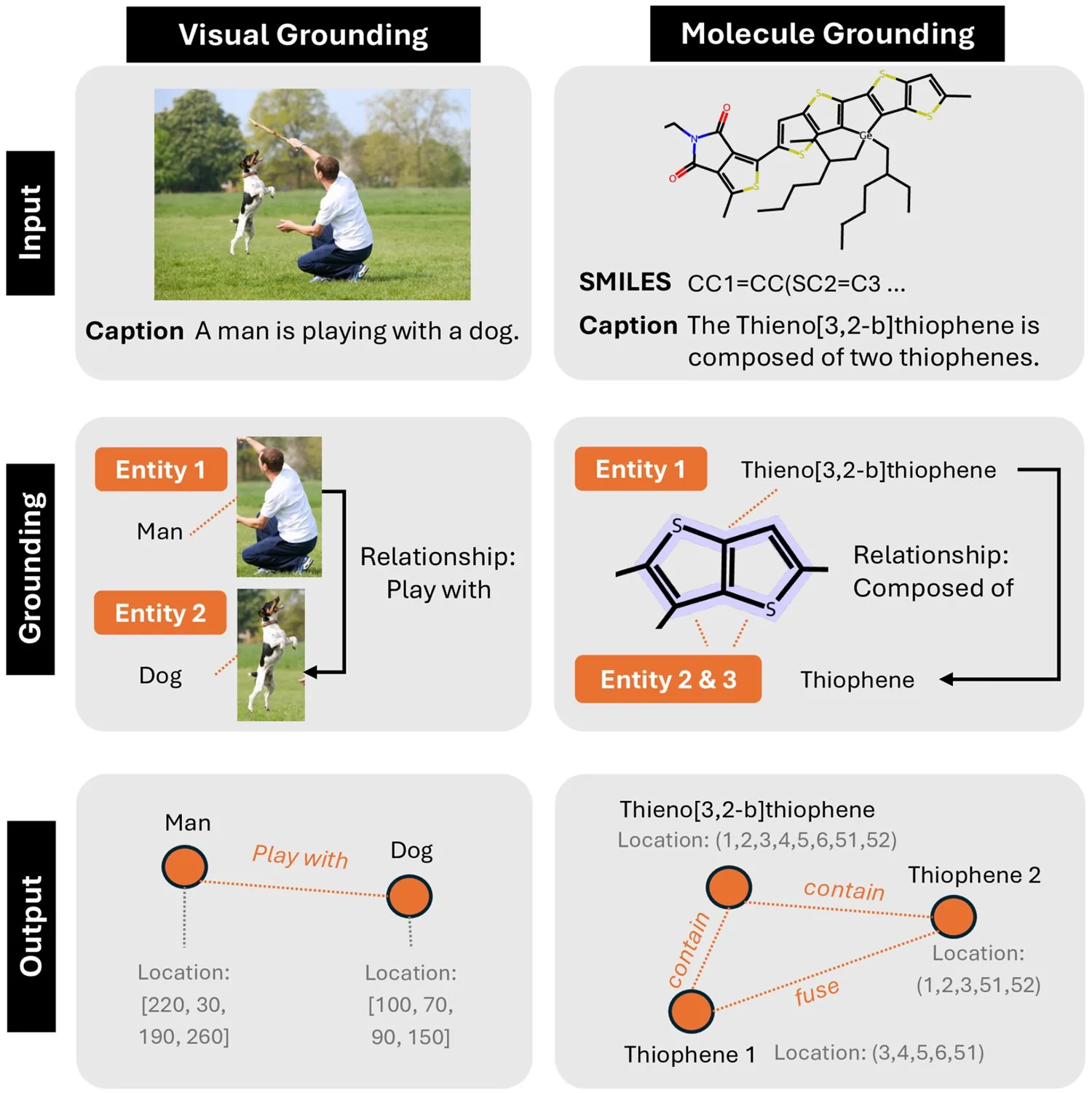
An illustration of the visual grounding and the molecular grounding task. Given a SMILES string and a descriptive caption, the model extracts the mentioned substructures, locates them within the SMILES representation, and builds a graph representing the relationships between them, which is similar to the visual grounding.

This challenge is analogous to the pivotal task of *visual grounding* in computer vision, which, by forcing models to link textual phrases to specific image regions, was instrumental in advancing vision-language models beyond high-level classification to a fine-grained, relational understanding of visual scenes. We posit that a similar leap is required to achieve true molecular intelligence, which is shown in Fig. 2. To catalyze this advancement, we introduce and tackle the task of *molecular grounding* (Wu *et al*. 2025): parsing a natural language description, identifying the mentioned molecular components, and constructing a relational graph that accurately reflects their positions and interactions within the formal chemical structure. However, unlike the pixel-based domain of images, grounding in the symbolic and graph-like domain of chemistry necessitates domain-specific reasoning capabilities. This requires translating a high-level caption and a low-level SMILES string into a structured, semantically rich graph.

**Figure 3:**
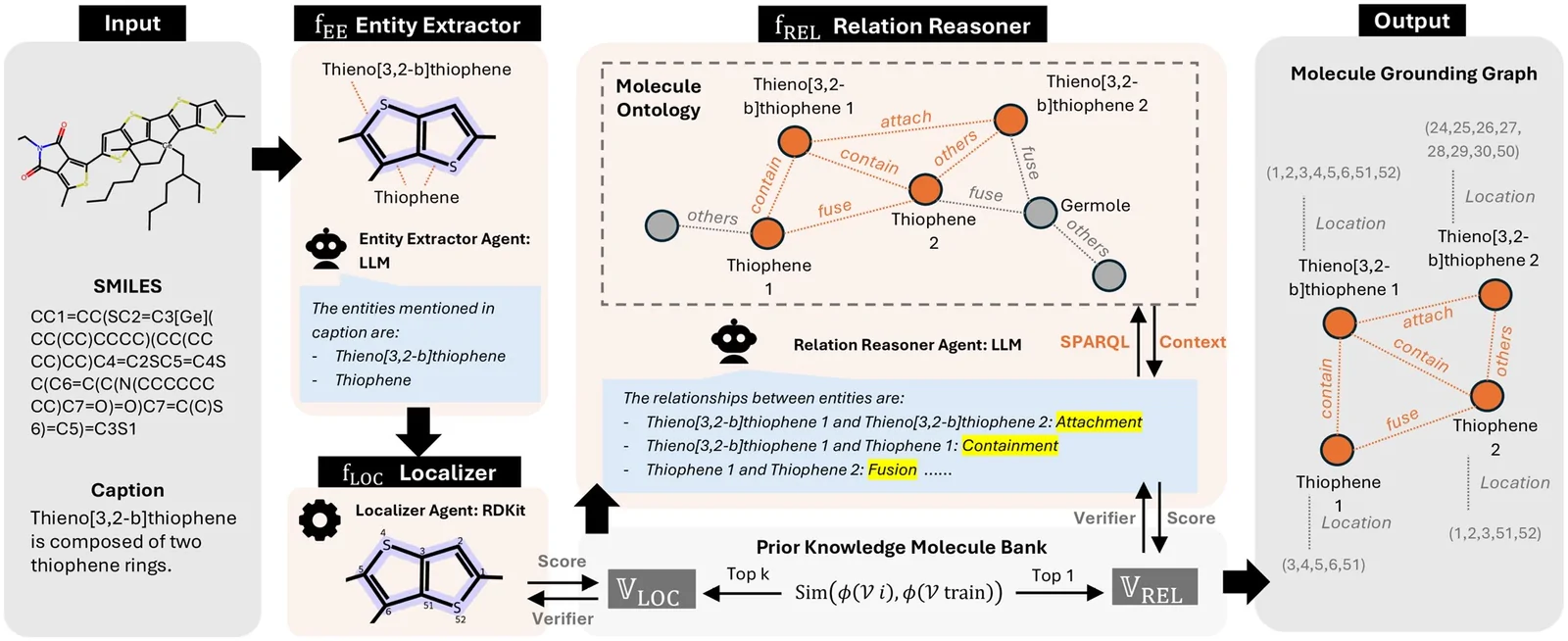
RelAgent is a multi-agent framework mimicking a human expert’s comprehension of the molecule grounding task.

Biologically, the goal of molecular grounding is to support tasks such as drug discovery (Chakraborty *et al*. 2023), patent interpretation (Jiang and Goetz 2025), and structural relationship analysis. Chemically, this requires recognizing functional groups, fused rings, linkers, and substituent relations inside a molecule (Liu *et al*. 2025). In particular, it can identify implicit relationships between substructures that are far apart in the molecular graph, which is important for molecule editing because modifying one region may affect another functionally related region. Computationally, it requires translating natural-language descriptions into a verified graph representation rather than relying on unconstrained end-to-end text generation.

In drug-design settings, such grounding is useful in at least two common scenarios: interpreting patent-style substructure descriptions in concrete candidate molecules, and supporting structure-constrained molecule editing where textual modification instructions must be mapped to exact molecular regions. In particular, it can identify implicit relationships between substructures that are far apart in the molecular graph, which is important because modifying one region may affect another functionally related region. Computationally, this requires translating natural-language descriptions into a verified graph representation rather than relying on unconstrained end-to-end text generation. Furthermore, fragment-based medicinal chemistry provides a concrete setting in which small entities are progressively elaborated into larger, more complex leads. Representative studies range from fragment-derived kinase inhibitors such as AT7519 and PF-06650833 to more recent experimental and computational fragment-elaboration workflows such as REFiL${\;}_{X}$, DeepFrag, and AIMLinker (Squires *et al*. 2009, Lee *et al*. 2017, Bentley *et al*. 2020, Green *et al*. 2021, Kao *et al*. 2023). These studies highlight that the practical challenge is not only where to add a fragment or linker, but also how to preserve the intended relationships between the retained core, the newly introduced substructure, and other functionally relevant motifs. In this context, RelAgent can serve as a complementary grounding and verification module for fragment-growing, scaffold-decoration, and molecule-editing systems.

However, achieving robust relationship grounding presents formidable challenges that render conventional end-to-end models ineffective. The core difficulties are threefold:

1. The Semantic-Structural Gap (Lee and Lim 2026): Natural language captions describe molecules using abstract, semantic concepts (e.g. “a benzene ring fused to a thiophene”), while the SMILES (Weininger 1988, O’Boyle 2012) string is a symbolic, sequential representation. Bridging this gap requires a model to map abstract entities onto specific, and often non-contiguous, segments of the SMILES sequence (Liao *et al*. 2024, Wu *et al*. 2025).
2. Complex Multi-Step Reasoning (Plaat *et al*. 2026 ): Grounding is not a single-step prediction. A human expert performs a sequence of cognitive tasks: first identifying the key entities, then locating them on the structure, and finally deducing their relationships. A monolithic model struggles to replicate this complex and hierarchical reasoning process, often failing at one of the intermediate steps (Ganeeva *et al*. 2024).
3. Implicit Chemical Knowledge (Zhang *et al*. 2025b): The relationships between substructures (e.g. fusion, linkage via a specific group) are governed by the strict rules of chemistry. This structured, domain-specific knowledge is often implicit in the caption and is not inherently known by general-purpose LLMs (Pei *et al*. 2024, Zhang *et al*. 2024), leading them to hallucinate chemically implausible connections.

Recent studies are closely related to our work but address different goals. MolGround mainly established a large benchmark for molecular grounding and highlighted the difficulty of fine-grained referential molecular understanding across multiple tasks (Wu *et al*. 2025). In contrast, our work focused on a concrete solution to the relation-centric grounding setting by predicting an explicit graph of substructures, localizations, and pairwise relationships. ChemGraph also adopted an agentic design, but it targeted computational chemistry workflow automation, including tool-augmented tasks such as SMILES conversion, geometry optimization, and thermochemistry calculations, rather than caption-to-structure grounding (Pham *et al*. 2026). MoleculeSTM learned a joint molecule-text representation for downstream tasks such as structure-text retrieval, text-based molecule editing, and molecular property prediction (Liu *et al*. 2023), but it did not address fine-grained grounding of textual mentions to localized substructures or reasoning over pairwise intra-molecular relations. Compared with these prior systems, RelAgent focused on fine-grained intra-molecular reasoning over the associations among multiple substructures, which is the central challenge of molecular relationship grounding.

To address the complexity of molecular grounding, any proposed solution must overcome three fundamental challenges. The first is *skill specialization*: the task requires a diverse set of abilities, where natural language understanding to parse text, spatial analysis (Li *et al*. 2025) to locate substructures, and logical reasoning to infer relationships (Plaat *et al*. 2026) . A single, monolithic model struggles to master all these distinct skills at once (Huang *et al*. 2026). The second challenge is *procedural flexibility*. Molecular grounding is not always a linear process; it can require iterative refinement (Hein *et al*. 2025), such as re-evaluating entity locations after failing to find a valid relationship. A rigid, static pipeline cannot accommodate such dynamic, back-and-forth reasoning. The third and most critical challenge is *interpretability and verifiability* (Tang *et al*. 2025, Naseem 2026). For a system to be trusted in a scientific context, its reasoning must be transparent (Zhang *et al*. 2025a). A single end-to-end “black-box” model, which conflates all reasoning steps, hides its decision-making process, making it impossible to debug or verify its scientific correctness (Pan *et al*. 2026).

We argue that a multi-agent framework provides a natural and effective architectural solution to these three challenges. To this end, we propose *RelAgent*, a cooperative multi-agent system designed for molecular grounding, where the baseline is shown in Fig. 3. RelAgent addresses the challenge of *skill specialization* by decomposing the problem into three distinct roles, each handled by a specialist agent: an Entity Extractor, a Localizer, and a Relationship Reasoner. It solves the need for *procedural flexibility* by enabling these agents to cooperate and be verified dynamically, allowing for goal-driven collaboration. Most importantly, RelAgent tackles the challenge of *interpretability* by design. Each agent’s output is an explicit, inspectable intermediate result, making the system’s overall reasoning process transparent and easy to debug. To further enhance verifiability, we introduce an ontology-guided mechanism that provides the Relationship Reasoner with formal, queryable chemical knowledge. Our entire framework is developed and evaluated on the MolGround benchmark (Wu *et al*. 2025), which provides the necessary task definition and dataset for this complex grounding problem.

Our contributions are as follows:

- We define and analyze the critical task of molecular grounding, identifying the core challenges that hinder existing models.
- We propose *RelAgent*, a novel multi-agent framework specifically designed to tackle molecular grounding by mimicking human-like, multi-step reasoning. On the MolGround benchmark, our method improved the vanilla LLaMA3.1-8B-Instruct’s (Touvron *et al*. 2023) baseline from 0.1% to 54.6% on REL F1 and achieved 56.0% exact-match F1 for localization, which was the strongest exact-localization result among the evaluated methods.
- We introduce a novel ontology-guided reasoning pipeline that constructs and utilizes a knowledge graph of molecular components, enabling the LLM to reason about complex structural relationships with high fidelity.

## 2 System and methods

### 2.1 Task formulation

We formally define the molecular grounding task as a system M that maps a descriptive input to a structured relational graph through a set of functional agents. The input is I=(S,C), where *S* is the molecule’s SMILES representation and *C* is the corresponding natural-language caption. Let $F={\left\{f_{i}\right\}}_{i=1}^{m}$ denote the set of specialized agents. The system outputs a predicted graph $G^{*}=(V,R)$, while $G_{\text{true}}$ denotes the ground-truth graph used for evaluation.


(1) $$M:(I,F)\to G^{*},\;I=(S,C),\;F={\left\{f_{i}\right\}}_{i=1}^{m},\;G^{*}=(V,R).$$

The vertex set V represents the unique molecular substructures that have been identified and localized. Each vertex is a tuple containing the entity name and its position within the SMILES string, and *k* is the number of identified substructure entities:


(2) $$V=\{(e_{1},l_{1}),(e_{2},l_{2}),\ldots ,(e_{k},l_{k})\},\;|V|=k,\;k\in Z^{+}.$$

The edge set R encodes pairwise relations between localized substructures. Each edge is a triplet $\left(v_{i},v_{j},\pi_{ij}\right)$, where $\pi_{ij}\in \Pi$ is a relation label from a predefined vocabulary Π (e.g. Fusion, Containment, or BondAttachment). In our formulation, the relationship reasoner examines each unordered pair of distinct vertices and assigns one relation label to that pair; therefore, the candidate graph contains at most $\left(\begin{array}{c} k\\ 2 \end{array}\right)$ pairwise edges:


(3) $$R=\{(v_{i},v_{j},\pi_{ij})|v_{i},v_{j}\in V,i<j,\pi_{ij}\in \Pi \},\;|R|\leq \left(\begin{array}{c} k\\ 2 \end{array}\right).$$

The primary objective of the framework is to select a predicted graph $G^{*}$ that best matches the ground-truth graph $G_{\text{true}}$. Our framework generates a candidate space containing multiple graph hypotheses, and the final output $G^{*}$ is the best-scoring candidate:


(4) $$G^{*}=\underset{G\in G}{\arg\;\min}\;L(G,G_{\mathrm{true}}).$$

Where G is the set of all candidate graphs produced for a given input. The loss function L is instantiated with various metrics to evaluate the accuracy of the predicted vertices and edges. In practice, our verification agents approximate this selection criterion. Detailed metrics are shown in Section Evaluation Metrics.

### 2.2 Multi-agent framework

Our multi-agent framework is designed to mirror the cognitive process a human expert follows when grounding a chemical caption to its molecular structure. This process logically decomposes into three stages: (1) reading the caption to identify all mentioned chemical substructures; (2) locating these substructures within the corresponding molecule; and (3) reasoning about the described relationships to assemble the final molecular graph. To operate this, we instantiate our agent set F with specialized components for Entity Extraction ($f_{\text{EE}}$), Localization ($f_{\text{LOC}}$), and Relationship Reasoning ($f_{\text{REL}}$). To manage the inherent ambiguity in this process and improve reliability, we employ a Best-of-N sampling strategy, where verifiers ($f_{\text{GND}}=\{V_{\text{LOC}},V_{\text{REL}}\}$) refine the candidate pool at critical stages.

The framework operates as a sequential pipeline. First, the *Entity Extractor* ($f_{\text{EE}}$) is prompted *n* times to identify relevant molecular entities from the caption *C*, producing a candidate pool E containing *n* distinct entity sets.


(5) $$\begin{array}{cc} f_{\text{EE}}:(S,C)\to E & \\ \;\;\;\;\text{where}\;E=\{E_{1},E_{2},\ldots ,E_{n}\},\;E_{i}=\{e_{1}^{i},\ldots ,e_{k_{i}}^{i}\} & \end{array}$$

Next, for each entity set $E_{i}\in E$, the *Localizer* ($f_{\text{LOC}}$) maps the entities to their character indices in the SMILES string *S*, yielding a collection of *n* candidate vertex sets, $C_{V}$. This pool is immediately filtered by the *Localization Verifier* ($V_{\text{LOC}}$), which selects the top-*p* most structurally plausible candidates (p≤n) to form a refined set, $C_{V}^{\prime }$.


(6) $$\begin{array}{c} f_{\text{LOC}}:(S,E_{i})\to V_{i},\;\text{producing}\;C_{V}=\{V_{1},\ldots ,V_{n}\}\\ V_{\text{LOC}}:C_{V}\to C_{V}^{\prime },\;\;\;\;\;\text{where}\;C_{V}^{\prime }=\{V_{1}^{\prime },\ldots ,V_{p}^{\prime }\} \end{array}$$

Finally, for each refined vertex set $V_{j}^{\prime }\in C_{V}^{\prime }$, the *Relationship Reasoner* ($f_{\text{REL}}$) infers the connections between vertices based on the caption, constructing a complete graph candidate, $G_{j}$. This results in a final pool of *p* graphs, $C_{G}$. The *Relational Verifier* ($V_{\text{REL}}$) then assesses this final pool and selects the single optimal graph $G^{*}$ as the system’s output.


(7) $$\begin{array}{c} f_{\text{REL}}:(S,C,V_{j}^{\prime })\to G_{j},\;\text{producing}\;C_{G}=\{G_{1},\ldots ,G_{p}\}\\ V_{\text{REL}}:C_{G}\to G^{*} \end{array}$$

This stage is executed pairwise over the refined vertex set. For each unordered pair of localized entities in $V_{j}^{\prime }$, the Relationship Reasoner queries the molecule-specific ontology constructed from the input SMILES to retrieve structural evidence, such as whether the two substructures are fused, connected by bond attachment, or in a containment relation. The agent then combines these retrieved facts with the caption semantics to assign a relation label $\pi_{ij}$ to the pair and assembles all pairwise labels into a graph candidate $G_{j}$. The Relational Verifier subsequently ranks the candidate graphs by global consistency with both the caption and the molecular structure, and selects the final output $G^{*}$.

## 3 Algorithm

### 3.1 Agent implementation

#### 3.1.1 Entity extractor

Mimicking the human ability to first identify key components in a description, the Entity Extractor ($f_{\text{EE}}$) serves as the entry point to our framework. This agent, a fine-tuned LLM, is responsible for parsing the caption *C* to identify all mentioned molecular substructures. For each of the *n* sample runs, the agent is prompted to generate a structured JSON object containing a list of entities and their corresponding SMILES strings. This process yields the candidate pool $E=E_{1},\ldots ,E_{n}$, and the prompt example is shown below:

$f_{EE}^{i}(S,C)$ : You are a chemistry expert. Given a caption {*C*} and SMILES {*S*} of a molecule, please extract its mentioned chemical entities from the caption where these entities exist in the given molecule, and output the chemical name with its SMILES. You should output the entities and their SMILESs into JSON format. If one entity is mentioned in the caption multiple times, you should output it once only.

To ensure the quality and relevance of these initial candidates, we enforce two critical constraints on each generated entity set $Ei=e_{1}^{i},\ldots ,ek_{i}{\;}^{i}$:

1. Semantic Relevance: Each extracted entity name must be explicitly present in the input caption *C*, ensuring the agent remains grounded in the provided text.
2. Chemical Validity: The SMILES string proposed for each entity must represent a valid chemical structure, a condition programmatically verified using RDKit.

These constraints prevent the model from hallucinating irrelevant or invalid structures, ensuring that the downstream agents operate on a high-quality foundation.

#### 3.1.2 Localizer and verifier

Following entity identification, the next logical step, akin to human spatial mapping, is to pinpoint these abstract substructures within the concrete SMILES string *S*. The Localizer agent ($f_{\text{LOC}}$) performs this task. For each candidate entity set $E_{i}$, it determines the precise 0-based character indices for every substructure SMILES, transforming the entity set into a vertex set Vi. This operation is applied across all *n* samples to produce the candidate collection $C_{V}$.

To enhance the robustness of this crucial step, the Localization Verifier ($V_{\text{LOC}}$) refines the candidate pool $C_{V}$ using a retrieval-based strategy grounded in prior knowledge. For each candidate vertex set $V_{i}$, we define a plausibility score. First, we retrieve the top-*K* most structurally similar molecules from our training data using Extended-Connectivity Fingerprints (ECFPs) (Rogers and Hahn 2010). The score for Vi is then calculated as the maximum cosine similarity between its embedding and the embeddings of the corresponding ground-truth localizations from the retrieved examples.


(8) $$\text{score}(Vi)=\text{Sim}(\phi (Vi),\phi (V{\text{train}}^{k}))$$

Where ϕ is a sentence embedding model. The verifier ranks all *n* candidates by this score and selects the top-*p* to form the refined set $C_{V}^{\prime }$, ensuring that only the most chemically plausible localizations proceed.

#### 3.1.3 Ontology-guided relationship reasoner and verifier

The final and most sophisticated cognitive step is to reason about the connections between localized components. Here, we face a basic problem: LLMs work by guessing based on language patterns, but chemistry follows strict, unbreakable rules. An LLM’s knowledge comes from statistics, not from a true understanding of chemical principles. This makes it unreliable for scientific tasks, as it can invent facts (a process often called “hallucination” (Xia *et al*. 2023)) and its reasoning is a black box, making it hard to trust or verify. Our core idea is to fix this by combining the strengths of both worlds: we use the LLM for its powerful language understanding, but we force it to base its reasoning on hard facts. To do this, we designed an *ontology-guided reasoning process*. This process takes the essential structural facts about a molecule and represents them outside the LLM in a clear, structured format, which is a knowledge base. The LLM-powered agent can then look up information in this knowledge base. This changes the agent’s task from guessing the relationship to interpreting the relationship based on provided facts.

**Figure 4:**
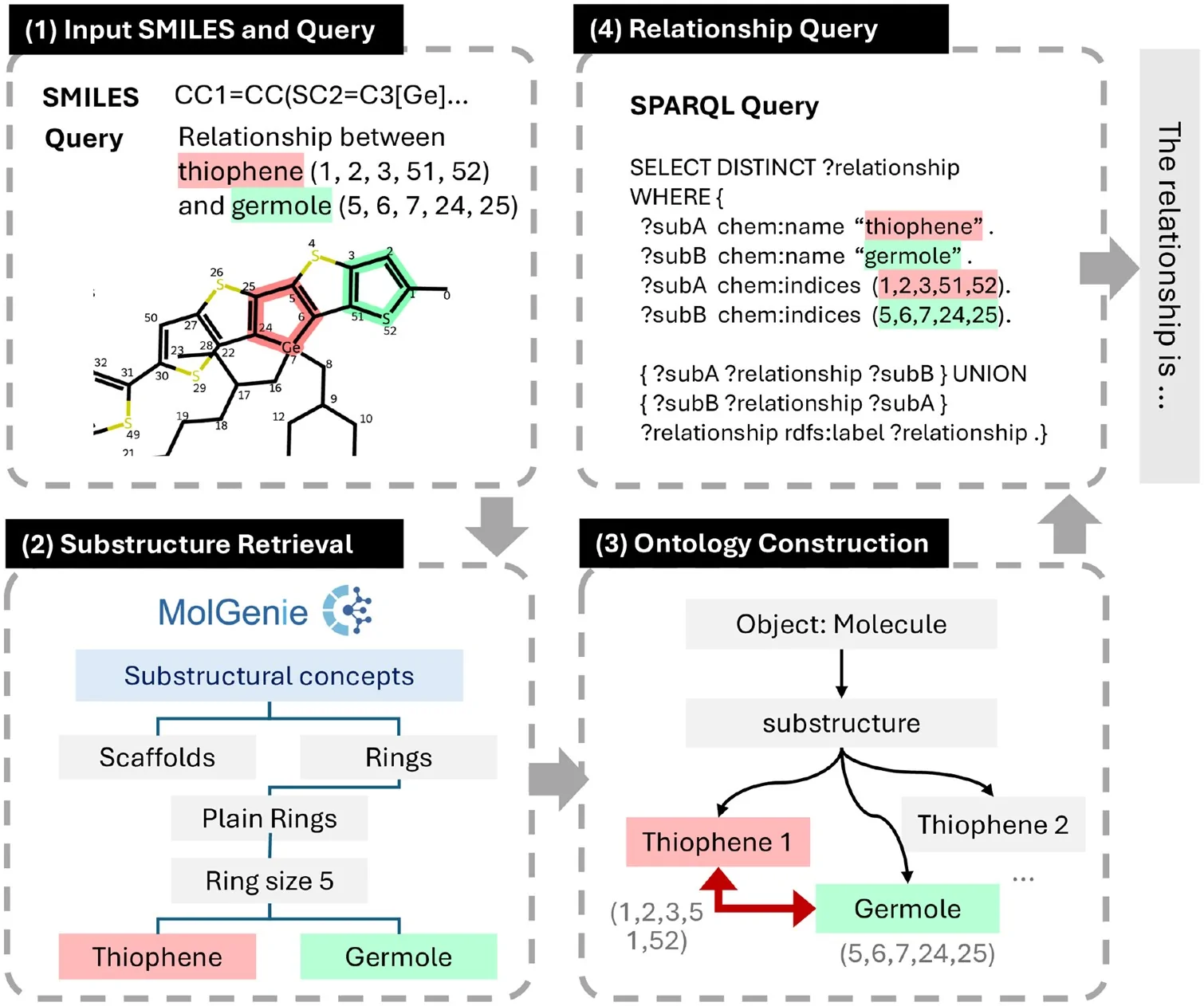
An illustration of our ontology-guided reasoning process. (1) For a given molecule (SMILES), we use MolGenie to extract all constituent substructures (e.g. Thiophene, Germole rings) and their atom indices. (2) This information is encoded into a dynamic, molecule-specific RDF knowledge graph (Molecular Ontology). Nodes represent substructure instances, and edges represent their relationships. (3) The Relationship Reasoner agent queries this ontology using SPARQL to retrieve factual relationships between specific entities, providing a strong structural prior for the final reasoning step.

To put this idea into practice, we dynamically construct a *Molecular Ontology* for each molecule. As shown in Fig. 4, this is not a general-purpose database but a custom-built knowledge graph *D* created on-the-fly from the SMILES string *S*. We use MolGenie (Krasnov *et al*. 2024) to parse *S* and break the molecule down into its substructures (e.g. scaffolds, rings). This information, including the exact atom indices for each part, is then stored using the Resource Description Framework (RDF). The result is a queryable graph where nodes are specific parts of the molecule (e.g. “Thiophene_1” at indices “(12,35,152)”) and edges are their well-defined chemical relationships (e.g. fusion, linkage). This turns the molecule’s hidden structural information into a clear, computer-checkable format.

The reasoning process is put into action by our Relationship Reasoner agent ($f_{\text{REL}}$). Instead of asking the LLM to infer connections from scratch, the agent acts as an information seeker. For a given pair of entities, the relationship module instantiated a SPARQL (Pérez *et al*. 2009) query template with the corresponding entity identifiers and atom indices, and executed it against the molecule-specific ontology graph D, as shown in Fig. 4. In this sense, the ontology was constructed dynamically for each molecule, while the querying procedure remained template-based and reproducible across samples. The query returned factual structural evidence, such as fusion, attachment, or containment, which was then inserted into the prompt for the reasoning LLM. This design reduced free-form hallucination and grounded the final relation prediction in explicit structural facts. The sample prompt is shown below:

$f_{\mathrm{REL}}^{i}(S,C,V_{j}^{\prime },\text{retrieved}\_\text{relations})$ : You are a chemistry expert. Based on the caption {*C*} and the provided structural facts {retrieved_relations}, determine the pairwise relationships for the entities {$V_{j}^{\prime }$}. The molecule is {*S*}. Provide the answer in JSON format.

This process generates the final candidate pool $C_{G}$. Finally, the Relational Verifier ($V_{\text{REL}}$) scores each complete graph $G_{j}$ based on its overall consistency, selecting the single highest-scoring graph $G^{*}$ as the final output.

## 4 Implementation

### 4.1 MolGround benchmark dataset

The MolGround dataset (Wu *et al*. 2025) is a comprehensive and rigorously constructed benchmark designed to advance the evaluation of molecular grounding tasks. It incorporates a relation-oriented approach to molecular understanding, focusing on the extraction, localization, and reasoning of substructures within molecules. The dataset was developed using an iterative methodology inspired by the Spiral Model (Boehm 1986), combining automated data structuring tools with extensive human expert validation. Data entries were sourced from authoritative molecular datasets, such as ChEBI-20 (Degtyarenko et al. 2007) and LPM-24 (Edwards *et al.* 2024), as well as chemical literature (Nagasawa *et al*. 2018). The final dataset encompasses 55 989 molecules, each paired with fine-grained captions that detail their substructures, stereochemistry, and conjugation systems. These captions were either directly sourced or generated using expert-curated templates, ensuring a consistent focus on molecular relationships and structural complexity.

To ensure the dataset’s reliability, rigorous annotation and cross-validation processes were employed. Seven annotators, supported by two independent expert reviewers, iteratively refined and validated the structured data to achieve consensus. The consistency of annotations was measured using Cohen’s kappa (McHugh 2012), achieving an exceptionally high agreement score of 0.985. This highlights the dataset’s quality and the robustness of its annotation process. Furthermore, the dataset covers a wide spectrum of molecular complexity, with atom counts ranging from 1 to 574 and bond counts spanning 0 to 642, capturing diverse structural features critical for grounding tasks.

### 4.2 Evaluation metrics

We evaluate the performance on the molecule grounding task using standard classification metrics: Precision, Recall, and F1 Score. These metrics are applied uniformly across Entity Extraction (EE), Localization (LOC), and Relationship Reasoning (REL) to provide a comprehensive assessment.

The metrics are defined as:


(9) $$\text{Precision}=\frac{\text{TP}}{\text{TP}+\text{FP}}$$

(10) $$\text{Recall}=\frac{\text{TP}}{\text{TP}+\text{FN}}$$

(11) $$F1\;\text{Score}=\frac{2\times \text{Precision}\times \text{Recall}}{\;\text{Precision}+\text{Recall}}$$

For Localization (LOC), we assess performance using both Exact Match (EM) and Intersection over Union (IoU) criteria:

*Exact Match (EM):* An exact match occurs when the predicted localization indices exactly match the ground truth indices.

*Intersection over Union (IoU):* For predicted localization spans $L_{p}$ and ground truth spans $L_{gt}$, IoU is calculated as:


(12) $$\text{IoU}=\frac{\left|L_{p}\cap L_{gt}\right|}{\left|L_{p}\cup L_{gt}\right|}$$

We report the following IoU-based metrics:

- *Average IoU:* The mean IoU over all matched localization spans.
  (13) $$\text{Average}\;\text{IoU}=\frac{1}{N}\sum_{i=1}^{N}{\text{IoU}}_{i}$$

Where *N* is the total number of matched spans.

- *Coverage* **:** The proportion of ground truth localizations that have at least one predicted localization with IoU exceeding a threshold τ:
  (14) $$\text{Coverage}=\frac{N_{\text{IoU}>\tau }}{N_{\text{gt}}}$$

Where $N_{\text{IoU}>\tau }$ is the number of ground truth spans with IoU above τ, and $N_{\text{gt}}$ is the total number of ground truth spans.

### 4.3 Experimental setup and baselines

The results of our experiments on the MolGround benchmark are summarized in Table 1. We compared RelAgent with a broad set of representative baselines spanning vanilla prompting, few-shot learning (Wang *et al*. 2020), supervised fine-tuning, retrieval-augmented generation (RAG) (Lewis *et al*. 2020), and long-reasoning chain-of-thought prompting (Wei *et al*. 2022). We expanded the baseline set to include both open-source models and stronger close-sourced proprietary models, including GPT-4o, GPT-5.3 (OpenAI 2026), Claude-Opus-4.6 (Anthropic 2026), and Gemini-3.1-Pro (Google DeepMind 2026).

**Table 1:** Comparison with representative baseline methods and frontier models on the MolGround benchmark.

Baseline	Backbone LLM	**EE**			**LOC (Exact Match)**			**LOC (IoU)**		**REL**		
		Prec. (%)	Recall (%)	F1 (%)	Prec. (%)	Recall (%)	F1 (%)	Avg. IoU (%)	Coverage (%)	Prec. (%)	Recall (%)	F1 (%)
Vanilla	GPT4o	72.1	78.8	73.5	1.4	1.2	1.2	11.4	26.5	28.1	14.4	16.0
	LLaMa3.1 8B	1.5	1.6	1.5	0.0	0.0	0.0	0.3	0.9	0.2	0.1	0.1
	LLaMa3.1 70B	6.1	6.7	6.2	0.1	0.1	0.1	0.7	2.0	0.5	0.7	0.4
	Claude-Opus-4.6	77.6	80.5	78.1	2.6	3.0	2.7	16.0	40.6	41.8	35.0	34.0
	Gemini-3.1-Pro	75.1	75.3	74.3	33.0	30.5	30.3	44.4	50.6	56.0	36.7	38.1
	GPT-5.3	68.4	70.9	68.7	6.1	6.4	6.2	15.5	31.5	31.7	20.3	21.9
Few-shot Learning	GPT4o	52.3	55.1	53.2	13.4	13.6	13.3	22.7	34.2	42.5	39.6	38.5
	LLaMa3.1 8B	28.1	30.2	28.7	8.5	8.3	8.3	12.8	17.8	21.5	20.3	19.5
	Claude-Opus-4.6	79.6	83.2	80.5	5.0	3.6	3.7	20.9	40.0	54.6	53.8	49.6
	Gemini-3.1-Pro	74.8	78.2	75.8	22.7	23.3	22.5	35.8	50.7	58.6	57.1	51.1
	GPT-5.3	77.1	81.1	78.3	5.3	5.4	5.1	18.3	36.3	50.5	44.6	43.0
SFT	LLaMa3.1 8B (MolGround SFT)	86.4	72.3	76.5	17.9	14.5	15.5	33.9	42.9	16.5	6.1	7.8
	Mol-Instructions (MolGround SFT)	59.7	49.1	51.4	10.6	7.8	8.8	25.1	26.6	18.5	5.6	7.2
Tool-Augmented LLM	CACTUS (LLaMa3.1 8B)	4.1	4.7	4.2	0.2	0.3	0.2	1.0	2.7	5.6	4.4	4.6
	ChatMol Copilot (GPT-5.3)	77.5	77.5	77.5	22.9	22.7	22.8	30.3	56.1	39.4	30.4	33.2
RAG	GPT4o	49.4	54.9	51.0	2.9	2.8	2.8	8.6	17.0	42.2	42.4	36.8
	LLaMa3.1 8B	25.9	28.5	26.7	1.4	1.0	1.1	5.5	7.6	13.5	10.7	9.2
	Claude-Opus-4.6	85.7	88.5	86.6	27.5	28.6	27.8	43.7	63.3	78.3	79.6	76.6
	Gemini-3.1-Pro	85.4	87.5	86.0	45.5	45.6	45.1	56.5	65.9	79.6	78.8	76.1
	GPT-5.3	87.8	90.1	88.5	23.9	24.0	23.8	39.3	56.1	74.4	71.0	69.0
Long-Reasoning CoT	LLaMa3.1 8B	65.0	66.5	64.4	0.7	0.8	0.7	11.3	26.6	12.8	5.9	6.4
	LLaMa3.2 1B	26.9	38.0	29.9	0.1	0.1	0.1	4.8	12.4	3.4	2.3	1.9
	Qwen2.5-7B	53.6	52.7	52.0	0.2	0.1	0.1	9.4	19.1	3.4	1.4	1.9
	DeepSeek-R1-Distill-Qwen-7B	46.7	50.4	46.5	0.4	0.2	0.3	8.9	22.1	9.1	3.5	3.7
	LLaMa3.1 8B (MolGround SFT)	70.2	62.2	64.0	7.7	5.4	6.0	22.4	36.0	12.2	4.7	5.7
	Mol-Instructions (MolGround SFT)	22.7	18.9	19.2	2.8	1.7	1.9	8.9	24.5	6.0	1.4	1.8
	Claude-Opus-4.6	71.7	72.4	71.0	3.9	2.5	2.7	17.9	32.8	46.5	40.5	38.8
	Gemini-3.1-Pro	69.8	67.4	67.4	16.4	16.3	15.6	26.9	44.0	50.6	53.6	49.3
	GPT-5.3	77.2	79.8	77.6	6.0	6.2	5.9	20.4	39.3	46.0	35.5	36.5
RelAgent (Ours)	LLaMa3.1 8B (EE + REL SFT)	81.2	82.3	81.4	56.3	57.1	56.0	57.5	61.1	62.2	56.9	54.6
	GPT-5.3	**90.0**	**88.5**	**89.0**	**67.4**	**74.9**	**70.2**	**68.4**	**84.6**	**82.5**	**80.9**	**79.8**

Metrics include precision, recall, and F1 score for Entity Extraction (EE), Localization (LOC), and Relationships (REL).

To dissect the contribution of each agent within our framework, we conduct a comprehensive ablation study. We begin with a vanilla baseline and incrementally add the Entity Extractor ($f_{\text{EE}}$), the Localizer ($f_{\text{LOC}}$), the Relationship Reasoner ($f_{\text{REL}}$), and finally the Grounding Verifiers ($f_{\text{GND}}$). The results are presented in Table 2.

**Table 2:** Ablation study of our proposed framework on the MolGround benchmark.

Baseline	**EE**			**LOC (Exact Match)**			**LOC (IoU)**		**REL**		
	Prec. (%)	Recall (%)	F1 (%)	Prec. (%)	Recall (%)	F1 (%)	Avg. IoU (%)	Coverage (%)	Prec. (%)	Recall (%)	F1 (%)
Vanilla	1.5	1.6	1.5	0.0	0.0	0.0	0.3	0.9	0.2	0.1	0.1
w $f_{\text{EE}}$	70.2	62.2	64.0	7.7	5.4	6.0	22.4	36.0	12.2	4.7	5.7
w $f_{\text{EE}}$, $f_{\text{LOC}}$	67.5	67.1	66.7	47.7	46.0	46.0	49.5	58.7	13.7	10.3	10.5
w $f_{\text{EE}}$, $f_{\text{LOC}}$, $f_{\text{REL}}$	80.5	81.8	80.7	**56.7**	**57.6**	**56.4**	**57.7**	**62.0**	61.0	55.8	53.6
w $f_{\text{EE}}$, $f_{\text{LOC}}$, $f_{\text{REL}}$, $f_{\text{GND}}$ (RelAgent)	**81.2**	**82.3**	**81.4**	56.3	57.1	56.0	57.5	61.1	**62.2**	**56.9**	**54.6**

We progressively add each agent module to a vanilla baseline to demonstrate its individual contribution to the overall performance.

### 4.4 Training details and reproducibility

Following the MolGround protocol with 1055 human-annotated molecules and, in total, 4.5k QA pairs derived from these molecules. We used the official train/validation/test split of 0.8/0.1/0.1 proportions. The Entity Extractor and Relationship Reasoner were fine-tuned from LLaMA3.1-8B using LoRA (Hu *et al*. 2022). The optimization setup used AdamW with learning rate 1.0e-4, batch size 8, and 3 epochs. We experiment on A100 hardware, with a total training cost of approximately 2 GPU-hours.

## 5 Discussion

### 5.1 RelAgent outperforms state-of-the-art methods

As shown in Table 1, RelAgent outperforms all baselines across almost all evaluation metrics, demonstrating its effectiveness in handling the molecule grounding task. Specifically, in the LOC (Exact Match) category, RelAgent achieves an F1 score of 56.0%, a massive improvement over the 15.5% from the best-performing baseline (LLaMa3.1 8B under SFT). This indicates that our method significantly improves the precision of localizing substructures. In the Relationships (REL) category, RelAgent achieves an F1 score of 54.6%, which is 16.1 absolute points higher than the second-best baseline (GPT4o under Fewshot). This highlights the effectiveness of our method in reasoning about complex relationships between molecular substructures, a critical capability for advancing referential molecular understanding.

The superior performance of RelAgent can be attributed to its fundamental architectural design. By integrating fine-tuned LLMs as specialized agents with structured molecular verifiers and ontology-based reasoning, it creates a robust, multi-step reasoning process. In contrast, monolithic approaches like vanilla and few-shot baselines struggle with consistency and precision, particularly in tasks requiring deep relational reasoning. Even fine-tuned models, while showing some improvements in specific sub-tasks, are limited by their inability to generalize and verify complex structural constraints, a gap that our agentic framework successfully fills.

### 5.2 Ablation study: deconstructing the agentic framework

**Figure 5:**
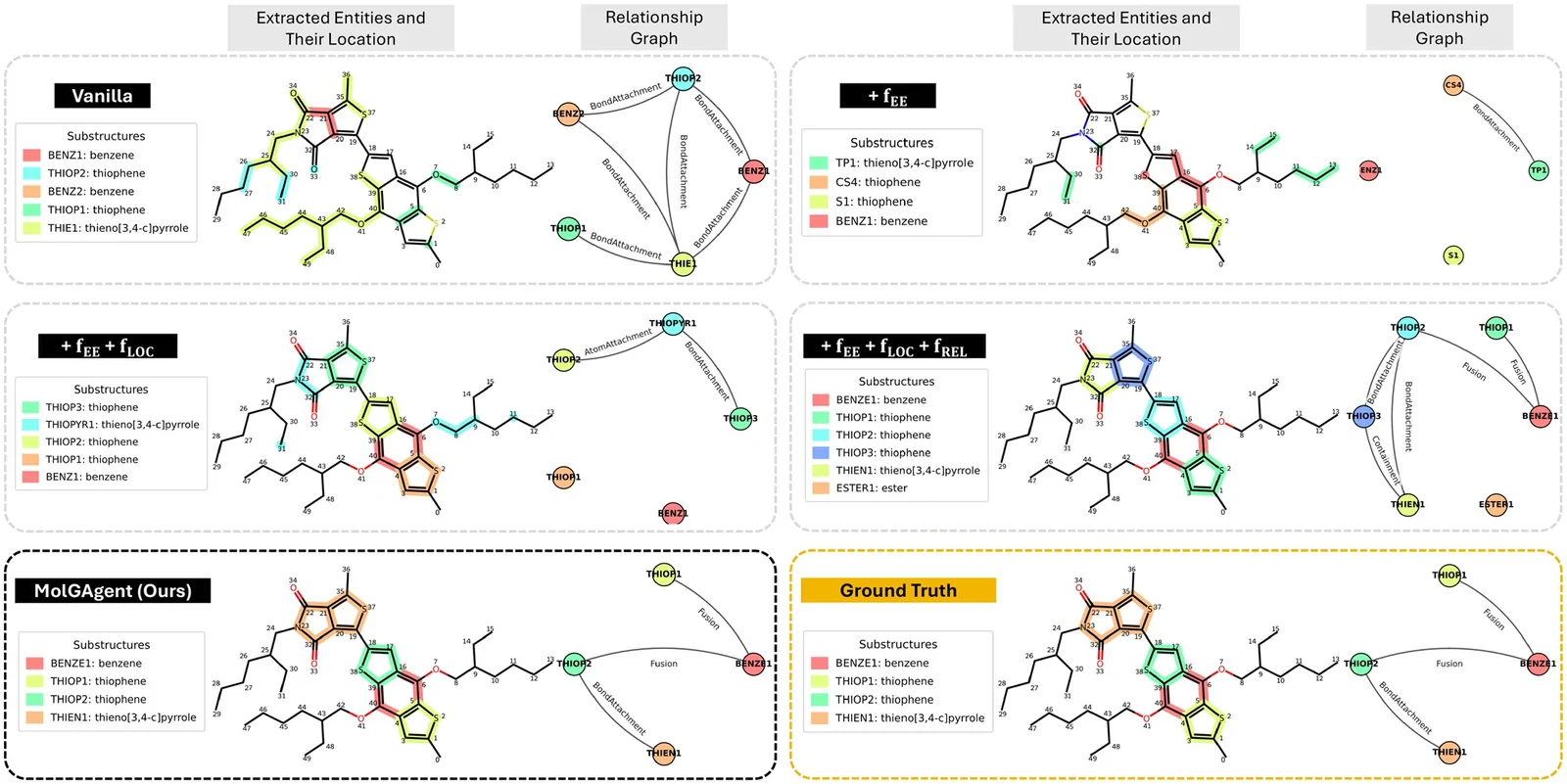
Qualitative ablation example on a complex MolGround test sample. Each row shows the extracted and localized substructures (left) and the resulting relationship graph (right) produced by progressively adding agents, from the vanilla baseline to the full RelAgent model. The example illustrates how $f_{\text{EE}}$ recovered relevant entity mentions, $f_{\text{LOC}}$ grounded them to specific structural positions, $f_{\text{REL}}$ assigned pairwise relation labels, and $f_{\text{GND}}$ selected the graph that best matched the ground truth. Graph labels and relation text were enlarged to improve readability.

Table 2 showed a clear stage-wise improvement pattern. Adding $f_{\text{EE}}$ mainly improved entity extraction (F1: 1.5% → 64.0%), confirming that reliable identification of mentioned substructures is the foundation of the task. Figure 5, provides critical insights into why the agentic architecture is necessary for this task. Each component’s contribution is not merely additive but synergistic, progressively building the capacity for complex reasoning. Adding $f_{\text{LOC}}$ then produced the largest gain in localization (Exact Match F1: 6.0% → 46.0%), demonstrating the importance of explicitly grounding textual entities to structural positions. Incorporating $f_{\text{REL}}$ yielded the major boost in end-to-end relational reasoning (REL F1: 10.5% → 53.6%), while the verifier module $f_{\text{GND}}$ provided the final refinement (REL F1: 54.6%). Although a few intermediate metrics fluctuated slightly due to stricter candidate filtering, the overall trend showed that each module contributed complementary information to the final grounded graph.

### 5.3 The foundational role of the entity extractor

The introduction of the Entity Extractor ($f_{\text{EE}}$) establishes a strong foundation, causing the EE F1 score to soar from a mere 1.5% to 64.0%. This highlights a core insight: general-purpose LLMs fail at even the first step of identifying key chemical components from text. A specialized agent is necessary. As seen in Fig. 5, the vanilla model hallucinates entities and produces chaotic outputs. In contrast, the model with $f_{\text{EE}}$ successfully identifies key entities like “thiophene rings,” providing a structured starting point. This demonstrates that problem decomposition is not just an optimization but a prerequisite for meaningful progress.

### 5.4 The critical need for a specialized localizer

Integrating the Localizer agent ($f_{\text{LOC}}$) boosts the LOC (Exact Match) F1 score from 6.0% to 46.0%. This confirms another key finding: abstract entity identification is useless without a mechanism to ground it in the physical structure (the SMILES string). The Localizer acts as a bridge between the semantic and structural worlds. Figure 5 visually confirms this, as the agent correctly highlights the atoms for each entity on the 2D diagram. This step transforms abstract names into concrete structural hypotheses, a crucial intermediate representation that monolithic models cannot produce.

### 5.5 The power of ontology-guided relationship reasoning

The most profound insight comes from the Relationship Reasoner ($f_{\text{REL}}$), which causes the REL F1 score to surge from 10.5% to 53.6%. This demonstrates that simply identifying and locating parts is insufficient; the true challenge lies in understanding their connections. Our key idea here is that leveraging a formal knowledge graph (ontology) as a structural prior allows the model to emulate sophisticated human reasoning, inferring complex relationships like “Fusion” and “Containment” that are implicit in the text.

Figure 5 provides a powerful illustration. The model with $f_{\text{REL}}$ correctly constructs the complex thieno[3,4-c]pyrrole core, a feat far beyond the previous steps. However, this stage also reveals a subtle but critical challenge: the model extracts a third thiophene ring (“THIOP3”) that, while structurally correct, violates the caption’s semantic constraint of “two thiophene rings.” This error is highly insightful, as it perfectly encapsulates the core tension between structural validity and semantic intent, underscoring the need for a final verification layer.

### 5.6 The Final Polish: verification as a refinement step

Finally, the inclusion of the verification agents ($f_{\text{GND}}$) provides the last piece of the puzzle, improving the final REL F1 score to 54.6%. The insight here is that multi-step reasoning processes can accumulate errors or generate multiple plausible hypotheses. The verifiers act as a crucial quality control and selection mechanism, filtering candidates to ensure the final output is not just structurally sound but also the most coherent interpretation of the initial prompt. In the case study, this final step resolves the ambiguity of the “third thiophene,” leading to the perfect ground truth match by selecting the interpretation that best aligns with all available information.

### 5.7 In-depth analysis of model capabilities and limitations

#### 5.7.1 Why standard LLM prompting fails at relational reasoning

**Figure 6:**
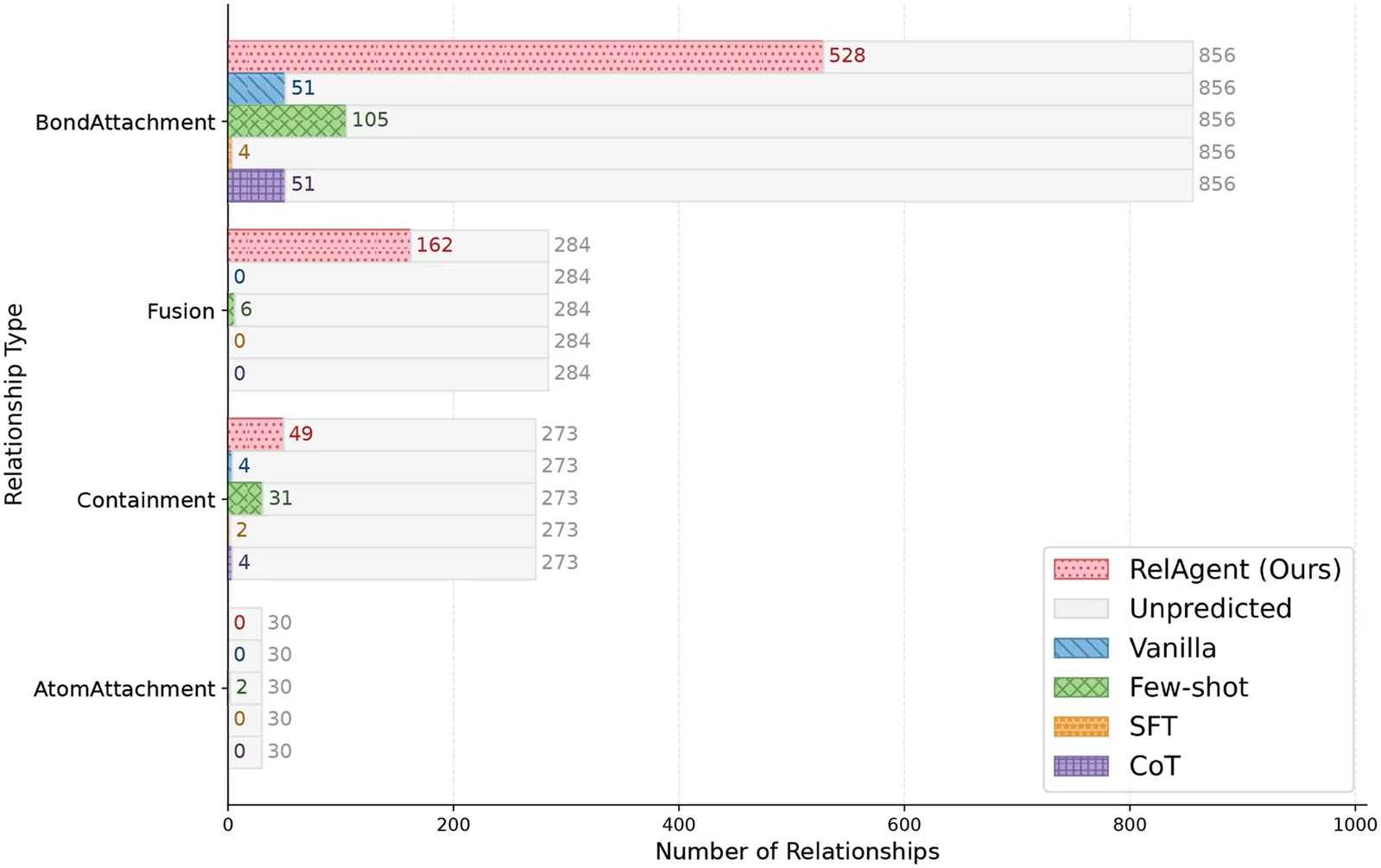
Relationship-type-wise prediction coverage across methods. For each relationship type, the colored bar shows the number of correctly predicted relations, while the gray remainder indicates the unpredicted portion relative to the ground-truth total. RelAgent substantially improves coverage on *BondAttachment*, *Fusion*, and *Containment*.

The relationship-type analysis in Fig. 6 makes the model’s strengths and failure modes more explicit. RelAgent correctly identifies 528 out of 856 *BondAttachment* relations, 162 out of 284 *Fusion* relations, and 49 out of 273 *Containment* relations, substantially outperforming all baselines. However, it still fails on *AtomAttachment* (0/30), a blind spot that is shared by nearly all compared methods.

This pattern suggests that the bottleneck is not merely one of general reasoning capacity, but also of representational granularity. Relations such as *BondAttachment*, *Fusion*, and *Containment* can often be inferred from substructure identities and their graph-level arrangement. In contrast, *AtomAttachment* requires identifying an exact atom-level attachment site, which demands finer-grained grounding than our current framework is designed to provide. In RelAgent, both the ontology construction and the reasoning process are primarily organized around substructures and their pairwise relations, rather than atom-centric attachment semantics. This likely explains why the framework remains strong on substructure-level relation reasoning but fails on this atom-level case. The rarity of *AtomAttachment* in the benchmark (30 instances) likely further compounds this difficulty.

#### 5.7.2 Agentic architecture confers robustness to long-range reasoning

**Figure 7:**
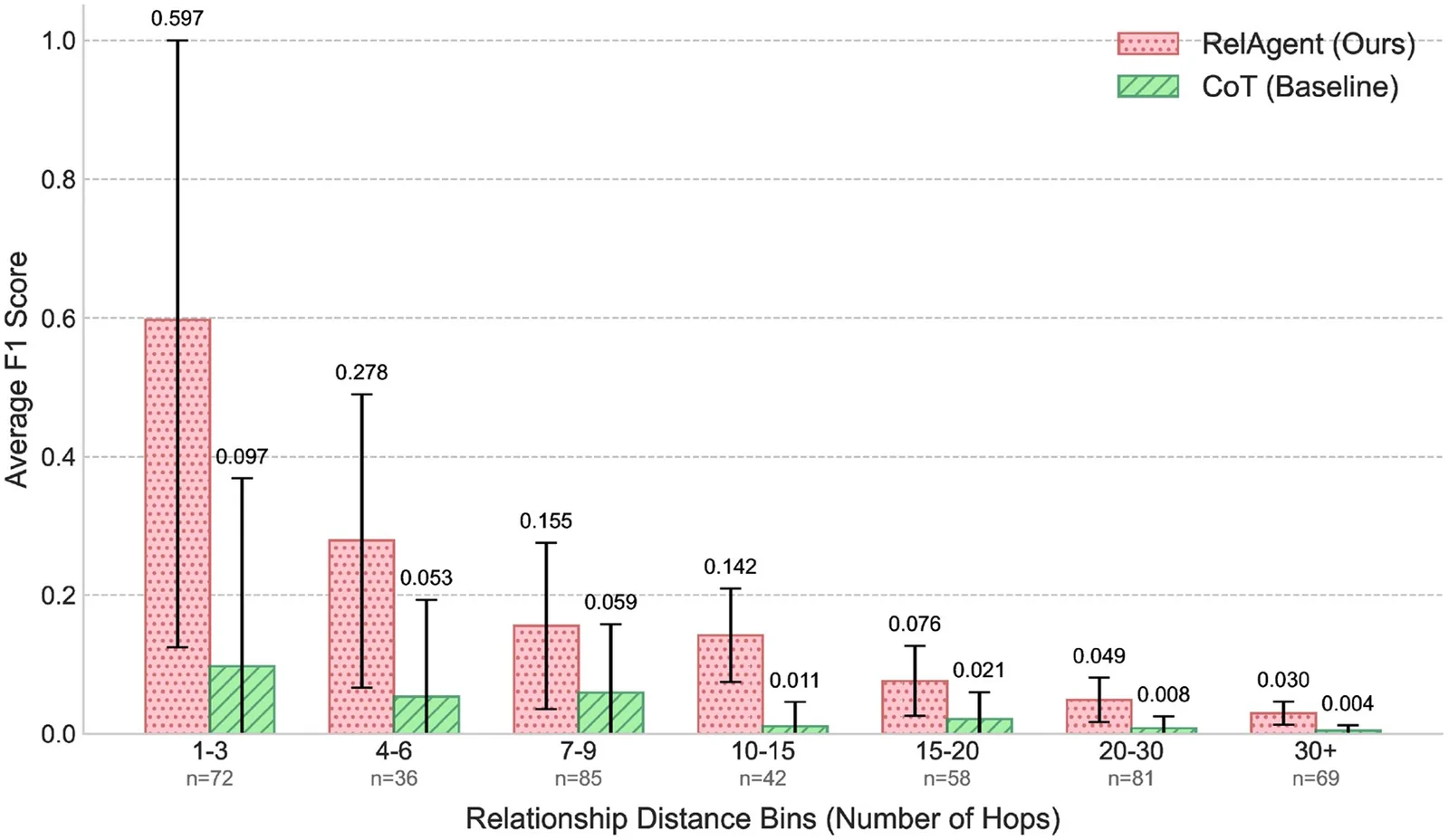
A fine-grained analysis of F1 scores for RelAgent and the CoT baseline, stratified by relationship distance. The distance bins are refined to better illustrate performance decay on long-range reasoning tasks. Error bars indicate standard deviation, and sample sizes (n) are provided for each bin.

The analysis of performance versus relationship distance (Fig. 7), which could be a proxy of the molecule complexity, provides strong evidence for the architectural superiority of our agent-based framework for complex reasoning.

RelAgent’s performance exhibits a graceful decay as the reasoning distance increases, maintaining a significant F1 score even for relationships spanning over 30 hops. In stark contrast, the performance of the monolithic CoT baseline collapses precipitously after 9 hops, becoming effectively useless for long-range connections.

This result offers a powerful insight: end-to-end reasoning chains are inherently fragile and susceptible to error propagation over long contexts. Our agentic paradigm, by decomposing the problem into verifiable, tool-augmented steps, effectively mitigates this failure mode. Each agent focuses on a smaller, more manageable part of the problem, and the use of verifiers prevents early errors from derailing the entire process. This demonstrates that for tasks requiring complex, multi-hop reasoning, an agentic architecture is not just incrementally better but qualitatively more robust and scalable.

#### 5.7.3 Potential applications for bioinformatics

Although RelAgent is not itself a molecule generator, it can serve as a complementary module in drug-design pipelines. For example, a medicinal chemist may specify a desired edit in natural language, such as preserving a fused heteroaromatic core while replacing a linker or terminal group. RelAgent can ground these instructions to explicit substructures, determine the relevant attachment or fusion relations, and convert them into structured constraints that are subsequently checked against candidate molecules. This makes the framework useful for patent interpretation, structural relationship analysis, and constraint verification in fragment-based or *de novo* molecular design.

## 6 Conclusion

In this paper, we introduced RelAgent, a multi-agent framework for molecular relationship grounding that decomposed a difficult caption-to-structure reasoning problem into interpretable stages of entity extraction, localization, and ontology-guided relationship reasoning. The results on MolGround demonstrated that this decomposition substantially improved both grounding accuracy and relational reasoning quality over vanilla prompting, retrieval-augmented, fine-tuned, and chain-of-thought baselines. Beyond benchmark performance, RelAgent is practically useful as a structure-aware reasoning layer for bioinformatics and medicinal chemistry applications. In particular, it can assist with patent interpretation, structure–activity relationship analysis, human-in-the-loop molecule editing, and downstream drug design workflows that require precise identification of the substructure referred to in text. Although RelAgent does not directly perform fragment assembly or de novo molecular generation, the grounded graph it produces can serve as a structured constraint interface for downstream generative, docking, or simulation systems. This makes RelAgent a promising component for more reliable and controllable AI pipelines in chemical and biomedical discovery.
